# Baseline predictors of neurophysiological response to combined physical and cognitive training in older adults with subjective cognitive decline

**DOI:** 10.1186/s12877-026-07270-8

**Published:** 2026-03-04

**Authors:** Hsien-Chun Chiu, Yu-Wei Hsieh, Chia-Hsiung Cheng

**Affiliations:** 1 School of Medicine, Chang Gung University, Taoyuan, Taiwan; 2 Laboratory of Brain Imaging and Neural Dynamics (BIND Lab), Chang Gung University, Taoyuan, Taiwan; 3 Department of Occupational Therapy and Graduate Institute of Behavioral Sciences, Chang Gung University, No. 259, Wenhua 1st Rd, Taoyuan, Taiwan; 4 Department of Physical Medicine and Rehabilitation, Chang Gung Memorial Hospital, Linkou, Taiwan; 5 Healthy Aging Research Center, Chang Gung University, Taoyuan, Taiwan; 6 Department of Neurology, Chang Gung Memorial Hospital, Kaohsiung, Taiwan

**Keywords:** Alzheimer’s disease, Subjective cognitive decline, Mismatch negativity (MMN), APOE ε4, physical and cognitive lifestyle, Multidomain intervention

## Abstract

**Background:**

Our previous study demonstrated that a 6-month combined physical and cognitive training program improved mismatch negativity (MMN)—an event-related potential reflecting the brain’s automatic detection of environmental changes—in older adults with subjective cognitive decline (SCD). However, not all individuals benefited equally from the intervention. Identifying baseline predictors of intervention outcomes could enable the prediction of individual responsiveness and support the development of personalized, stratified intervention strategies to promote brain health in this at-risk population. This study aimed to identify baseline predictors of response to a 6-month combined cognitive and physical training program among older adults with SCD.

**Methods:**

We conducted a secondary analysis of a cluster-randomized trial involving 33 older adults with SCD who participated in twice-weekly integrated training sessions. MMN was assessed for the analytic sample (*n* = 21) as the primary outcome measure. Baseline predictors included age, general cognitive function, physical and cognitive activity engagement, and apolipoprotein E (APOE) genotype. Linear regression analyses were performed to examine predictors of change in MMN amplitude from baseline to post-intervention.

**Results:**

Regression analysis indicated that APOE ε4 non-carriers showed significantly greater improvement in MMN amplitude (β = ‒0.449, *p* = 0.027). Additionally, lower baseline levels of physical activity demonstrated a trend toward association with greater MMN gains (β = ‒0.392, *p* = 0.050), suggesting that individuals with more sedentary lifestyles may derive greater benefit from the intervention.

**Conclusions:**

APOE ε4 carrier status and baseline physical activity levels may be associated with the effectiveness of multidomain interventions in older adults with SCD.

**Supplementary Information:**

The online version contains supplementary material available at 10.1186/s12877-026-07270-8.

## Introduction

Subjective cognitive decline (SCD) refers to an individual’s self-perceived worsening of cognitive abilities—particularly memory—despite performing within normal limits on standardized cognitive assessments [1–3]. Clinically, SCD is important because it may represent the earliest stage of neurodegenerative processes [4, 5]. Accumulating evidence indicates that individuals with SCD are at increased risk of subsequent progression to mild cognitive impairment (MCI) and Alzheimer’s disease (AD) dementia [6, 7], highlighting SCD as a clinically relevant construct for studying the earliest stages of AD–related cognitive change. Given this elevated risk, early intervention in individuals with SCD is critical, as it may delay cognitive deterioration or reduce the likelihood of progression to MCI or dementia.

Cognitive decline is influenced by a range of biological and behavioral factors. Accordingly, multidomain interventions—typically combining physical exercise, cognitive training, and lifestyle modifications—are often recommended [8–10]. However, evidence regarding their effectiveness in individuals with SCD remains mixed. For example, the Finnish Geriatric Intervention Study to Prevent Cognitive Impairment and Disability (FINGER) trial reported improved memory performance among SCD participants following a two-year multidomain lifestyle intervention that included dietary guidance (3 individual sessions and 7–9 group sessions), cognitive training (10 group sessions and 144 individual sessions [10–15 min per session, 3 sessions per week]), and physical activity (1–3 sessions per week for muscle strength training; 60–90 min per session, 2–5 sessions per week for aerobic training) [11]. In contrast, the Protein Omega-3 aNd vitamin D Exercise Research (PONDER) study focused on dietary supplementation and exercise. The exercise program comprised one-hour exercise sessions twice-weekly, with 10 min of aerobic exercise and 30–40 min of progressive resistance training. However, this study found no significant cognitive differences between intervention and control groups after six months [12]. Our previous randomized controlled trial (RCT) involved six months of combined physical and cognitive training in older adults with SCD. The intervention protocol consisted of twice-weekly, supervised sessions where 1 h of physical exercise was immediately followed by one hour of cognitive training. The physical component integrated resistance training using Thera-Bands and water bottles, balance training, and aerobic activities. While the primary neuropsychological outcome—a composite Z-score derived from the Chinese Version Verbal Learning Test, Logic Memory Test, Complex Figure Test, Digit Span Backward, Trail-Making Test B, and the Cognitive Abilities Screening Instrument—did not show significant group differences, neurophysiological assessments revealed enhanced brain plasticity, as indicated by changes in mismatch negativity (MMN) [13]. These findings suggest that neural adaptations may emerge prior to detectable improvements in cognitive performance.

Mismatch negativity (MMN) is an event-related potential (ERP) component that reflects the brain’s automatic response to deviations in repetitive sensory input [14–16]. By examining MMN amplitude and latency, researchers can assess pre-attentive auditory processing and cortical plasticity, making MMN a valuable neurophysiological marker for evaluating intervention effects. While our prior work showed intervention-related enhancements in MMN [13], the variability in individual outcomes highlights the need to identify baseline predictors of response. Pinpointing these predictors would allow for a more personalized approach to intervention, improving its overall efficacy and reducing unnecessary efforts for non-responders. Moreover, understanding these predictive factors can illuminate the mechanisms by which multidomain interventions exert neurophysiological effects in older adults with SCD.

Several factors have been proposed as potential predictors of intervention outcomes, including age, baseline cognitive function, APOE genotype, and lifestyle behaviors. Age, in particular, is frequently identified as a key determinant of intervention efficacy. Younger individuals often exhibit greater cognitive improvements than older adults. For instance, Salminen et al. (2016) found that although both younger and older adults benefited from dual n-back working memory training, the younger group showed a greater magnitude of improvement in working memory performance as measured by mean n-back level [17]. Similar findings have been reported in clinical populations; for example, Ophey et al. (2021) observed that younger individuals with Parkinson’s disease achieved larger verbal and visual-spatial working memory gains following 5 weeks of computerized adaptive working memory training [18]. In cognitively intact older adults, several studies have shown that older age is associated with smaller improvements following cognitive training [19, 20]. Despite this body of evidence, few studies have examined whether age moderates the effects of integrated physical and cognitive training specifically in the SCD population.

Baseline cognitive performance is another consistent predictor of training outcomes. Zinke and colleagues reported that individuals with lower baseline performance on working memory tasks showed the greatest improvements in executive control, verbal, and visuospatial working memory following home-based adaptive, process-based working memory training [20], suggesting that those with more room for improvement may derive more benefit. Similar trends have been observed in physical exercise interventions. For example, it has been found that participants with lower baseline scores on the Montreal Cognitive Assessment (MoCA) showed greater cognitive and physical improvements following dual-task training programs that combined physical movement with cognitive tasks (HAPPY program), as measured by gains in MoCA and the Short Physical Performance Battery. The program lasted 3 months with twice-weekly, 1-hour exercise sessions [21]. These findings suggest that baseline cognitive status may play a key role in determining responsiveness to both cognitive and physical interventions.

Genetic factors, particularly the presence of the apolipoprotein E epsilon-4 (APOE ε4) allele, may also influence intervention outcomes. Carriers of the APOE ε4 allele have a significantly elevated risk of developing MCI and dementia—estimated at 2.6 times higher than non-carriers [22, 23]. It has been reported that non-carriers with greater left hippocampal volume exhibited larger gains in semantic verbal fluency following cognitive training, suggesting a potential interaction between genetic and neuroanatomical factors in predicting intervention response [19]. Moreover, López-Higes and colleagues conducted multidomain cognitive training in cognitively normal older adults, and the results indicated that in the domain of language comprehension (specifically complex sentence processing), significant training benefits were observed only in APOE ε4 non-carriers, whereas APOE ε4 carriers did not exhibit noticeable improvements. These findings suggest that genotype moderates the efficacy of cognitive training, with non-carriers deriving greater benefits [24].

Engagement in healthy lifestyle behaviors, including more frequent participation in physical and cognitive activities prior to intervention, has long been associated with a reduced risk of cognitive decline [25–27]. Previous literature, based on the similar group of subjects as in our study, reported that older adults without MCI nor dementia undergoing structured cognitive training with lower baseline cognitive performance demonstrated significantly greater improvements in language memory, attention, and executive function compared to those with higher baseline performance [28]. However, very few studies have investigated whether these pre-existing behaviors predict responsiveness to multidomain interventions. It remains unclear, for instance, whether individuals with more sedentary lifestyles may show greater intervention-related gains due to a larger scope for improvement, or conversely, whether more cognitively or physically active individuals may benefit more due to higher baseline plasticity.

The primary objective of this study is to identify baseline predictors of neurophysiological response to an integrated physical and cognitive training intervention in community-dwelling older adults with SCD. Building on prior literature, we hypothesize that younger age, lower baseline cognitive performance, and the absence of the APOE ε4 allele will be associated with greater post-intervention improvements in MMN amplitude. Although few studies have explored the role of pre-intervention lifestyle in moderating treatment response, we further hypothesize that individuals with more sedentary lifestyles will exhibit larger MMN changes, reflecting greater neuroplastic potential. By integrating demographic, cognitive, genetic, and behavioral predictors, this study aims to advance precision intervention strategies for older adults at risk of cognitive decline.

## Methods

### Participants

This study is a secondary analysis of data from our previous cluster-randomized trial (ClinicalTrial ID: NCT04162990), which implemented a 6-month multimodal intervention combining physical exercise and cognitive training for older adults with SCD [13].

Eligible participants were community-dwelling older adults aged 50 years or older. The lower age threshold was selected to capture individuals at the earliest at-risk stage of SCD, which has been shown to emerge during late midlife prior to overt cognitive impairment. Following the operational definition of SCD, participants were required to report a self-experienced persistent decline in cognitive abilities compared with their status two years prior. This was screened using a 12-item SCD questionnaire, where inclusion required a “yes” response to at least one item, along with confirmation of the decline by a family member or close friend [14, 29]. To ensure the sample represented a pre-MCI stage, participants had to demonstrate objective cognitive performance within the normal range, specifically achieving a score of > 79 on the Cognitive Abilities Screening Instrument (CASI).

Exclusion criteria were strictly applied to ensure data integrity; individuals were excluded if they or their first-degree relatives had been diagnosed with mental diseases. Further exclusions included a history of severe neurological diseases, brain injury leading to loss of consciousness, or cardiovascular diseases. Participants with a history of alcohol, nicotine, or substance dependence, as well as those suffering from visual or hearing degeneration (including hearing aid users), were also excluded.

A total of 50 participants who met the criteria were enrolled (intervention group: *n* = 33; control group: *n* = 17). For the present secondary analysis, which aimed to identify predictors of intervention outcomes, only data from the intervention group were included. At baseline (T1), 33 participants in the intervention group completed assessments. Following the 6-month intervention, 22 participants completed the post-intervention assessment (T2). One dataset was excluded due to poor data quality, resulting in a final analytic sample of 21 participants. In the present sample, the mean age was 64.82 ± 1.00 years, with the majority of participants aged 60 years or older.

### Procedure

Prior to the intervention, participants underwent neuropsychological assessments, neurophysiological recordings, and blood sample collection (T1). Demographic information (e.g., age, sex, education) and self-reported lifestyle habits (e.g., cognitive and physical activity routines) were also collected. Blood samples were used to determine APOE ε4 genotype status. Neuropsychological and neurophysiological assessments were repeated immediately after the intervention (T2) and again at six months post-intervention (T3). This secondary analysis focused specifically on identifying predictors of immediate intervention effects (i.e., changes from T1 to T2).

The intervention consisted of twice-weekly sessions, each lasting two hours, over six months (total: 48 sessions). Each session included one hour of physical exercise incorporating resistance, balance, and aerobic training and one hour of cognitive training targeting attention, processing speed, memory, and executive function, conducted in small groups of 4 to 12 participants. Two licensed occupational therapists, trained specifically for this program, delivered the intervention. Further details of the training protocol and intervention content are described in our previous study [13].

### ERP recording and pre-processing

ERPs were acquired using a dry-electrode, battery-powered, portable DSI-24 EEG headset (WEARABLE Sensing Inc., San Diego, USA). The system included 19 electrodes distributed according to the international 10–20 system, along with two electrodes at the bilateral earlobes. Eye blinks and movements were monitored using electrooculograms (EOG) placed above the left orbit and below the right orbit. All signals were online referenced to Pz, with a sampling rate of 300 Hz, and electrode impedance was maintained below 1 MOhm.

Data processing was performed using EEGLAB software. The offline ERP data were re-referenced to the average of the two earlobes (A1 and A2) and filtered with a bandpass of [0.1, 30] Hz. Independent Component Analysis (ICA) was applied to identify and remove ocular artifacts. Additionally, epochs with activity exceeding ± 75 µV on the channels of interest were excluded from averaging.

### Mismatch negativity (MMN)

MMN is an event-related potential reflecting the brain’s automatic response to auditory irregularities. It was elicited using an auditory oddball paradigm consisting of frequent standard tones (1000 Hz, probability = 0.85) and infrequent deviant tones (900 Hz, probability = 0.15), with an inter-stimulus interval (ISI) of 1000 ms [30, 31]. Participants passively listened to the auditory stimuli while watching a silent, emotionally neutral video with subtitles. The ERP data were segmented into 600-ms epochs, including a 100-ms pre-stimulus baseline. ERPs were averaged separately for standard and deviant stimuli, and the MMN waveform was obtained by subtracting the standard response from the deviant response (i.e., deviant minus standard). To ensure comparable signal-to-noise ratios between conditions, only standard trials immediately preceding deviant trials were selected, resulting in a similar number of trials for standards and deviants.

The MMN peak amplitude and peak latency were determined as the most negative deflection occurring between 100 and 300 ms at the Fz electrode, where the MMN response is typically maximal. On average, approximately 116 to 119 deviant trials were utilized to estimate the ERP waveforms for each participant across measurements.

### Cognitive abilities screening instrument (CASI)

CASI is a comprehensive tool designed to assess global cognitive function across nine domains, including memory, attention, orientation, language, visual construction, abstraction, judgment, and fluency. Its validity has been demonstrated in multiple studies: CASI shows adequate ecological, convergent, and discriminative validity in people with dementia and significant ability to distinguish between dementia patients and controls [32]. Psychometric analyses confirm CASI’s unidimensional construct and satisfactory reliability, with Rasch analysis supporting its use for overall cognitive assessment and demonstrating measurement consistency across genders [33].

### Blood sample and APOE ε4 genotyping

To determine APOE ε4 carrier status, DNA was extracted from white blood cells in blood samples. Specific regions of the APOE gene were analyzed to detect polymorphisms at codons 112 and 158, which indicate the presence of the ε4 allele, a known genetic risk factor for AD [34].

### Assessment of physical and cognitive activity routines

#### Physical activity

Participants also reported engagement in physical activities at three intensity levels, each including seven examples: (1) light intensity: morning stretches, low-intensity ball games (e.g., croquet), slow dancing, yoga, fishing, walking or dog walking, light housework; (2) moderate intensity: hiking, moderate-intensity ball sports (e.g., badminton), casual cycling or swimming, dancing, martial arts, heavy housework; (3) vigorous intensity: running, high-intensity ball games (e.g., soccer, basketball), mountain/stair climbing, fast cycling, lap swimming, aerobic/energetic dancing, jump rope.

Physical activity scores were calculated using the same frequency-duration system as for cognitive activities [35, 36]. To account for intensity, a weighting factor was applied: light activities × 3, moderate activities × 5, and vigorous activities × 9, as their corresponding anticipated metabolic equivalent values [37, 38]. These weighted scores were summed to generate the total physical activity score.

#### Cognitive activity

Participants reported their engagement in 10 common cognitive activities, including: (1) watching movies, (2) listening to music or radio, (3) reading newspapers or books, (4) playing card games, (5) playing puzzle games, (6) using computers or phones (e.g., browsing, video watching, online chatting), (7) playing online or mobile games, (8) visiting museums or attending performances, (9) participating in religious or temple events, and (10) engaging in handicrafts.

Cognitive activity levels were quantified using a frequency-duration scoring system. Participants received points based on how often they engaged in each activity (1 = almost never; 3 = 1–2 times per month; 5 = 1–2 times per week; 9 = more than 3 times per week) [37]. Additionally, duration scores were assigned based on the average time spent per session (1 = less than 1 h; 3 = 1–2 h; 5 = 2–4 h; 9 = more than 4 h). The total cognitive activity score was calculated by multiplying the frequency and duration scores for each activity and summing the scores across all 10 activities [35, 36].

### Statistical analysis

Numerical data are presented as mean ± standard error of the mean (SEM). To reduce the risk of Type I errors and address potential violations of parametric assumptions, we conducted Spearman rank correlation analyses between the change in MMN amplitude (∆T2–T1) and potential predictor variables, including age, physical activity score, cognitive activity score, and baseline general cognitive performance (measured by CASI). We also conducted a Mann–Whitney U test to compare changes in MMN amplitude (ΔT2–T1) between APOE ε4 carriers and non-carriers.

Only variables showing significant bivariate associations with MMN change (*p* < 0.05) were retained for subsequent regression analyses. To evaluate whether these variables predicted the efficacy of the intervention, we used linear regression models, with the immediate change in MMN amplitude (∆T2–T1) as the dependent variable. Predictor variables included physical activity score, and APOE ε4 genotype (coded as 0 = non-carriers, 1 = carriers). A backward stepwise method (using default settings) was applied to identify significant predictors. The results regarding model fit, including R², adjusted R², and the overall F-test of the regression model were reported. In addition, post-estimation diagnostic analyses were conducted to examine key assumptions of linear regression, including multicollinearity (i.e., variance inflation factor, VIF), residual normality, and influential observations (leverage values and Cook’s distance).

All statistical analyses were performed using SPSS version 19.0 (IBM Corp., Armonk, NY, USA). The alpha level for statistical significance was set at 0.05, except for the backward stepwise regression, where an alpha level of 0.10 was used for variable removal [39].

## Results

### Baseline characteristics

The baseline characteristics of participants in the intervention group are summarized in Table 1. A total of 33 older adults with SCD, but without detectable cognitive impairment, were included in this predictor analysis.

**Table 1:** Baseline characteristics of the intervention group

Characteristic	Intervention (*n* = 33)
Age	64.82 ± 1.00
Sex (male/female)	8/25
Education	13.64 ± 0.56
APOE4 (carrier/non-carrier)	8/25
History of hypertension (yes/no)^a^	4/29
History of diabetes (yes/no)^a^	5/27^b^
CASI	93.94 ± 0.72
SCD score	4.39 ± 0.47
Cognitive activity score	105.33 ± 14.03
Physical activity score	536.33 ± 62.87
Total lifestyle score	641.67 ± 68.11
GDS	1.42 ± 0.24

Data are presented as mean ± standard error of the mean (SEM), unless otherwise specified. *APOE*4 Apolipoprotein E4, *CASI* Cognitive Abilities Screening Instrument, *SCD *Subjective cognitive decline, *GDS *Geriatric Depression Scale

^a^ All the participants who reported a history of hypertension or diabetes were controlled by medication

^b^ One subject’s information was missing

### Correlation analysis

**Fig. 1:**
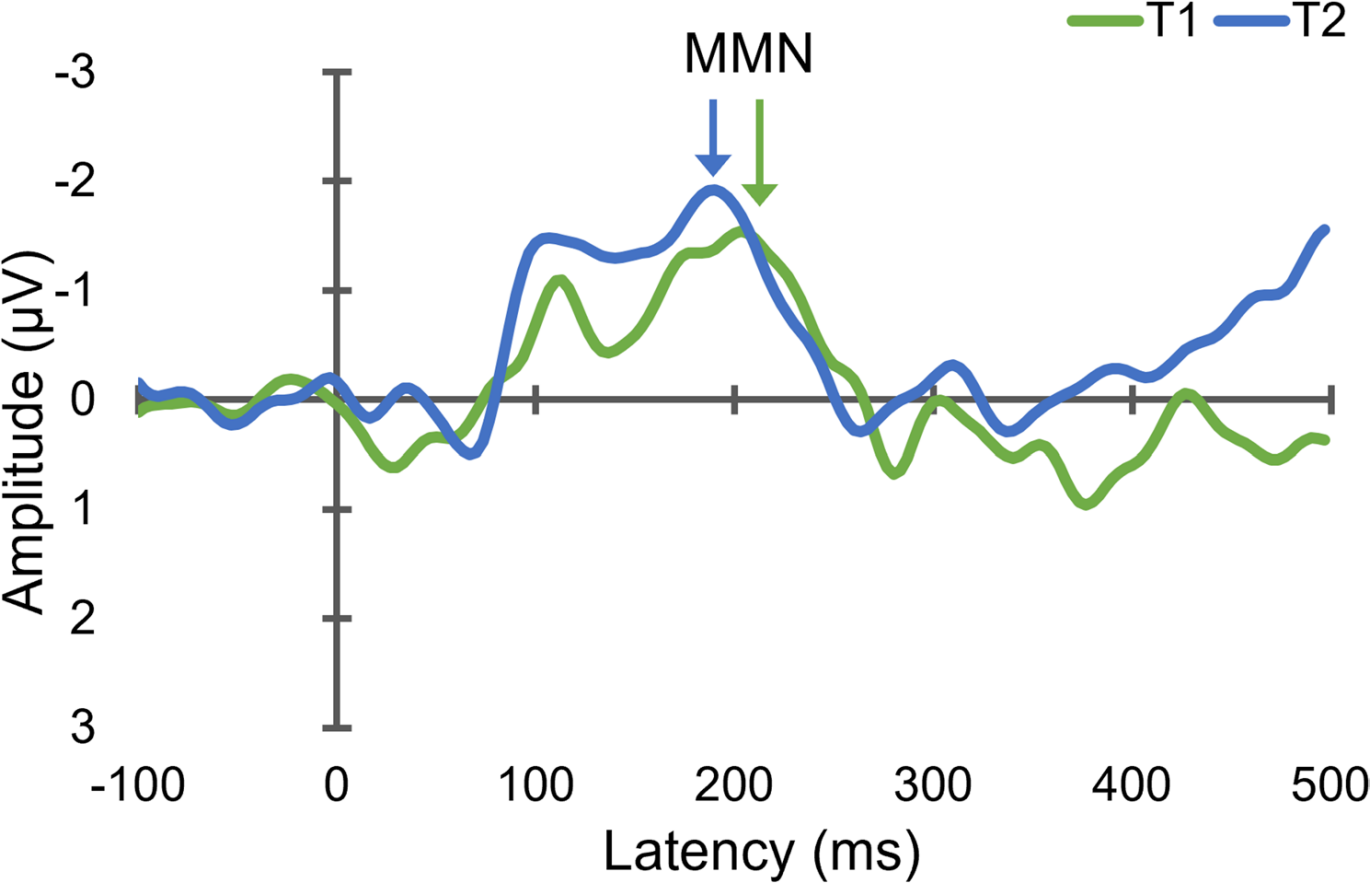
Grand-averaged mismatch negativity (MMN) waveforms at the Fz electrode are shown for the pre-intervention (T1) and post-intervention (T2) assessments in participants who received combined physical exercise and cognitive training. MMN was derived by subtracting event-related potentials (ERPs) elicited by standard stimuli from those elicited by deviant stimuli (i.e., deviant minus standard). Waveforms are time-locked to stimulus onset (0 ms), with negative voltage plotted upward. The displayed epoch includes a 100-ms pre-stimulus baseline. Visual inspection suggests enhancement in MMN amplitude following the intervention, reflecting improved automatic auditory change detection after training

Figure 1 displays the grand-averaged MMN responses obtained before and after the intervention. Guided by previous literature, we examined five potential predictors of training-related improvements in MMN amplitude: age, CASI score, APOE ε4 genotype, physical activity score, and cognitive activity score. Among these, physical activity score showed a moderate-to-strong correlation with training-related changes in MMN amplitude (*p* = 0.021). Additionally, the APOE ε4 genotype was significantly associated with changes in MMN amplitude (*p* = 0.036). The results are summarized in Table 2 and Supplementary Fig. 1.

**Table 2:** Correlations and p-values between baseline characteristics and MMN amplitude improvement (ΔT2–T1)^a^ after intervention

*R*	Cognitive activity score	Physical activity score	APOE4	Age	CASI
	-0.088	-0.5	--	-0.144	0.119
p value	0.705	0.021*	0.036*	0.533	0.606

*MMN *Mismatch negativity, *R *Spearman rank correlations, *APOE4 *Apolipoprotein E4, *CASI *Cognitive Abilities Screening Instrument, *T1 *Baseline, *T2 *End of the intervention

^a^ MMN is conventionally characterized by a negative-going waveform, with more negative peak amplitudes reflecting stronger pre-attentive auditory processing. For clarity and consistency in statistical interpretation, MMN amplitudes were transformed to absolute values prior to analysis, such that larger values uniformly indicate greater neural responsiveness. This approach facilitates intuitive interpretation of model coefficients and avoids ambiguity when relating intervention effects to changes in neurophysiological function

*P < 0.05

### Regression analysis

Variables showing significant bivariate associations with MMN change (*p* < 0.05) were entered into a multiple linear regression model. As shown in Table 2, APOE ε4 genotype, and physical activity score were entered for subsequent regression analyses.

The overall regression model including APOE ε4 status and baseline physical activity score was statistically significant (F_2,18_ = 5.37, *p* = 0.015), indicating that the combination of these predictors significantly explained variance in MMN amplitude change. Model fit indices showed an R² of 0.374 and an adjusted R² of 0.304, with a standard error of the estimate of 1.449. Assessment of multicollinearity indicated no violations of model assumptions. The VIF values for both predictors were 1.003 (tolerance = 0.997), well below commonly accepted thresholds, confirming the absence of collinearity concerns. Normality of residuals was supported by the Shapiro–Wilk test (*p* = 0.215), indicating that unstandardized residuals did not deviate significantly from a normal distribution. Leverage diagnostics showed that although a small number of observations exhibited relatively higher leverage (centered leverage values ranging from 0.029 to 0.497), the Residuals vs. Leverage plot did not show any cases combining high leverage with large residuals. Notably, the high-leverage observation was accompanied by a small residual, consistent with a “good leverage point” that aligns with the fitted regression pattern rather than an influential aberration. Consistently, all Cook’s distance values (range = 0.000 − 0.303) were well below the conventional threshold of 0.5, indicating that neither the high-leverage cases nor the identified outlier exerted undue influence on the regression estimates. In addition, studentized residuals ranged from − 1.673 to + 1.708, remaining within widely used criteria for outlier screening (absolute studentized residual ≤ 2). Within this model, APOE ε4 non-carriers exhibited significantly greater improvements in MMN amplitude compared to ε4 carriers (β = −0.449, *p* = 0.027). Baseline physical activity score showed a borderline association with MMN change (β = −0.392, *p* = 0.050), suggesting that individuals with lower baseline physical activity tended to demonstrate greater neural plasticity gains following the intervention (Table 3).

**Table 3:** Multiple regression analysis of predictors for MMN amplitude improvement following intervention

Variable	Improvement of MMN amplitude (∆T2-T1)^a^				
	B	95% CI for B	β	*P*	VIF
Constant	2.464	0.962 to 3.966		0.003	--
Physical activity Score	-0.002	-0.005 to 0.000	-0.392	0.050^#^	1.003
APOE4 carriers	-1.569	-2.938 to -0.199	-0.449	0.027*	1.003

B Unstandardized regression coefficients, *CI *Confidence interval, *β *Standardized regression coefficients, *VIF *Variance inflation factor

^a^ MMN is conventionally characterized by a negative-going waveform, with more negative peak amplitudes reflecting stronger pre-attentive auditory processing. For clarity and consistency in statistical interpretation, MMN amplitudes were transformed to absolute values prior to analysis, such that larger values uniformly indicate greater neural responsiveness. This approach facilitates intuitive interpretation of model coefficients and avoids ambiguity when relating intervention effects to changes in neurophysiological function

*p < 0.05

#*p* = 0.05

## Discussion

This study is among the first to explore predictors of cognitive and physical training outcomes in older adults with SCD. Specifically, we examined factors predicting improvements in MMN amplitudes after a 6-month integrated cognitive and physical training intervention. Our analysis identified APOE ε4 genotype as a significant predictor of MMN improvement, with APOE ε4 non-carriers exhibiting more substantial gains in MMN amplitudes compared to APOE ε4 carriers. Additionally, lower previous exercise engagement demonstrated borderline significance, suggesting potential greater intervention-related neurophysiological benefits in individuals less physically active at baseline.

Our findings reinforce prior evidence that APOE ε4 carrier status significantly impacts the cognitive benefits derived from interventions. APOE ε4 is well-documented as a risk factor for cognitive impairment [22, 23, 40]. Previous studies have highlighted that APOE ε4 carriers experience attenuated cognitive improvements from lifestyle interventions compared to non-carriers. Solomon et al. (2018) reported a subgroup analysis from the FINGER trial, indicating diminished cognitive intervention efficacy in APOE ε4 carriers [41]. Further research also showed APOE ε4 non-carriers with higher baseline hippocampal volumes had greater training gains from cognitive interventions [19], aligning with our findings of greater MMN amplitude improvements in APOE ε4 non-carriers. As for the possible underlying mechanism, APOE ε4 carriage has been associated with reduced cholinergic function, potentially impairing synaptic plasticity and neuronal efficiency required for cognitive improvements [42, 43]. It was further interesting to note that the intravenous injection of scopolamine, a drug with central cholinergic antagonist, would reduce MMN amplitude to frequency deviants as recorded by magnetoencephalography in a sample of 13 healthy adults as compared to the delivery of glycopyrrolate, a drug with only a peripheral anticholinergic modulation [44]. Collectively, these findings indicate that the effectiveness of multimodal interventions in SCD may be influenced by APOE genotype.

Our results identified baseline exercise engagement as another potentially relevant predictor of MMN improvements. Although exercise engagement exhibited only borderline statistical significance (*p* = 0.050), it merits discussion given existing literature on exercise and cognitive health. Previous studies have consistently shown that physical exercise positively impacts cognitive health in older adults through enhanced neurogenesis, synaptogenesis, increased cerebral blood flow, and elevated neurotrophic factor levels [25, 45–47]. Studies in animal models also showed that voluntary exercise accelerates the integration of new neurons into existing neural circuits, enhancing cognitive function and hippocampal plasticity [48]. The above literature explained the mechanisms of progress in cognitive performance after training, and may also explain why lower previous exercise participation benefited more after the training, since there were more new neurons left to be integrated. This finding also reflected a ceiling effect. Our findings suggest that individuals already engaged in consistent high-intensity exercise may derive comparatively smaller benefits from additional interventions than those with lower baseline activity levels. This pattern is consistent with prior clinical research [20, 49]. In addition to the study discussed in the Introduction [20], David and colleagues also reported that participants with lower baseline fitness (VO_2_max) experienced greater cognitive and neurophysiological improvements following a structured multicomponent exercise intervention. Specifically, individuals with poorer baseline fitness levels demonstrated larger gains in executive functions and structural preservation of AD-related brain regions, such as the hippocampus, further demonstrating that baseline physical fitness or activity levels may significantly moderate the effectiveness of cognitive and physical interventions [49]. Taken together, our findings suggest that individuals with sedentary lifestyles should participate in active physical and cognitive training more since they have greater potential to benefit from cognitive and physical interventions. Future similar studies can consider our findings and incorporate a preliminary screening process to identify individuals with lower previous physical activity levels prior to cognitive and physical interventions.

Although previous studies have consistently reported that younger individuals tend to exhibit greater cognitive improvements following training interventions [17, 18, 20], our analysis did not identify age as a significant predictor of neurophysiological gains. Two factors may explain this discrepancy. First, the participants in the present study were community-dwelling older adults with SCD who were cognitively intact and relatively homogeneous in age (mean 64.82 ± 1.00 years). This restricted age range likely reduced between-subject variability and the statistical power to detect age-related differences. Second, our multimodal intervention combining physical and cognitive training, rather than sole cognitive training, could have mitigated age-related differences in neural responsiveness. Physical exercise is known to upregulate neurotrophic factors, particularly brain-derived neurotrophic factor (BDNF) and insulin-like growth factor-1 (IGF-1), and to enhance cerebral blood flow, thereby promoting neuroplasticity even in advanced age. Meta-analyses of randomized controlled trials in older adults demonstrate that both aerobic and resistance exercise significantly increase circulating BDNF and IGF-1 levels, with moderate-to-vigorous intensity and longer duration interventions yielding the largest effects [50, 51]. These neurotrophic factors are essential for synaptic plasticity, neurogenesis, and neuronal survival, and their elevation is associated with improved cognitive function and memory performance in aged populations [52, 53].

Contrary to previous findings suggesting that baseline cognitive performance predicts the magnitude of training-related gains [20, 28, 54], our analysis did not identify baseline global cognition, as measured by the CASI, as a significant predictor of neurophysiological improvement. Several factors may explain this observation. First, the participants in the present study were cognitively intact older adults with SCD and thus exhibited minimal variability in baseline cognitive scores (mean CASI = 93.94 ± 0.72). This homogeneity likely limited the ability to detect predictive effects. Second, our outcome measure—MMN amplitude—indexes pre-attentive auditory processing rather than explicit, higher-order cognitive performance [16]. However, the CASI primarily reflects global cognitive status and may not sensitively capture domain-specific abilities [55, 56]. Consequently, individuals with higher or lower baseline cognitive function may exhibit comparable neurophysiological adaptability. Third, multimodal interventions integrating physical and cognitive components might attenuate the moderating role of baseline cognition, as physical exercise has been shown to enhance neurotrophic signaling and neural efficiency across the baseline cognitive status [57, 58]. Together, these factors suggest that within a relatively high-functioning SCD population, the capacity for neural plasticity—as reflected by MMN changes—may be more strongly determined by biological or lifestyle factors than by global cognitive status at baseline.

Several limitations of the present study should be acknowledged. First, although the study targeted older adults with SCD, the minimum age criterion was set at 50 years in order to capture individuals at the earliest at-risk stage of SCD, which may emerge during late midlife prior to overt cognitive impairment. As a result, the age range of the sample may limit the generalizability of the findings to more advanced geriatric populations. Notably, however, only three participants in the present sample were younger than 60 years, with the majority of participants falling within the age range typically considered geriatric. Nevertheless, future studies with larger samples and stricter age thresholds are warranted to determine whether the identified baseline predictors of neurophysiological responses remain applicable in older and more clinically impaired groups. Second, the small sample size limits the generalizability of our findings and may reduce statistical power, increasing the risk of both Type I and Type II errors. Although the observed trends are promising, replication in larger and more diverse cohorts is needed to confirm the identified predictors of neurophysiological change. Third, while the primary aim of the present study was to examine the direct associations between the selected baseline predictors and the neurophysiological outcome, the potential influence of other mediating or moderating factors beyond the identified predictors in the observed associations remains an important consideration. Our study design and sample size were not specifically powered to support formal mediation or moderation analyses. Fourth, the use of MMN as the sole outcome measure limits the scope of interpretation. While MMN is a sensitive and objective marker of cortical plasticity, it does not necessarily translate into real-world functional or cognitive improvements. Fifth, the cross-sectional nature of the predictor analysis precludes definitive conclusions about causality. Although associations between baseline characteristics and MMN changes were identified, the observed relationships may be influenced by unmeasured confounding variables, such as sleep quality, mental health, or social engagement, which were not assessed in this study. Finally, self-reported lifestyle behaviors, including physical and cognitive activity levels, may be subject to recall bias or social desirability bias. While the frequency-duration scoring system offers a structured approach, more objective measures such as actigraphy could improve accuracy in future studies. Furthermore, our assessment of cognitive lifestyle did not account for the varying cognitive demands or difficulty levels of different activities, which may influence neuroplastic potential differently.

In conclusion, APOE ε4 status was associated with cognitive and physical intervention efficacy in older adults with SCD, as measured by improvements in MMN amplitude. Previous exercise involvement was also a possible indicator. These findings emphasize the necessity for personalized intervention strategies, tailored according to genetic predisposition and initial lifestyle factors, to maximize cognitive health benefits in aging populations.

## Supplementary Information

Supplementary Material 1.

## Data Availability

Data available on request from the corresponding author with reasonable reasons.
