# Supplementary material for: Baseline predictors of neurophysiological response to combined physical and cognitive training in older adults with subjective cognitive decline
Source: BMC Geriatr. 2026 Mar 4;26:507. doi: 10.1186/s12877-026-07270-8 (PMC13067679; doi:10.1186/s12877-026-07270-8)
Supplement: Supplementary file 1 — Supplementary Material 1. [file 12877_2026_7270_MOESM1_ESM.docx]

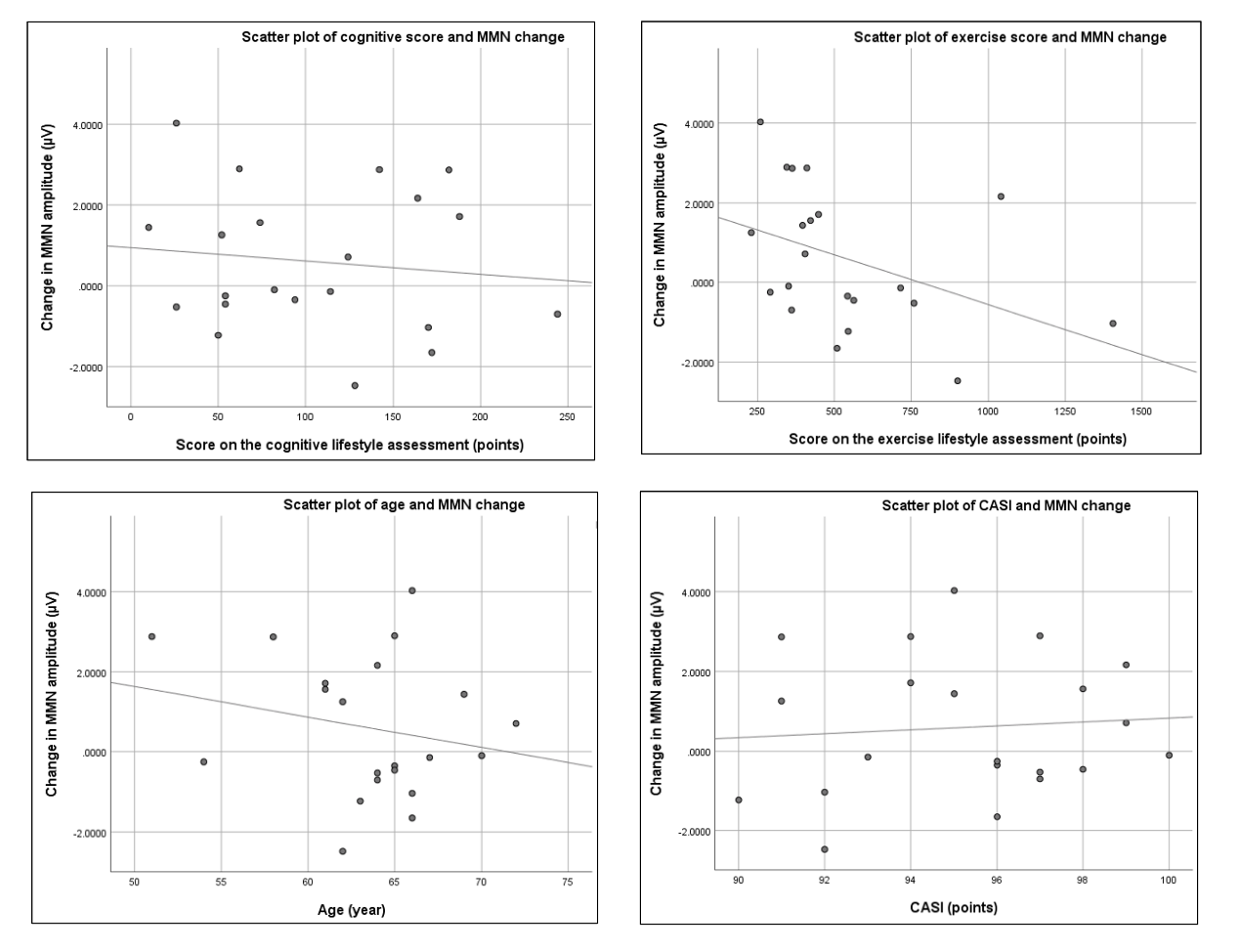


**Supplementary Figure 1**. Scatter plots illustrating the associations between baseline characteristics (cognitive activity score, physical activity score, age, and CASI score) and changes in MMN amplitude following the intervention.

CASI = Cognitive Abilities Screening Instrument; MMN = mismatch negativity
